# Maternity Care Access and Infant Mortality

**DOI:** 10.1001/jamanetworkopen.2025.42831

**Published:** 2025-11-11

**Authors:** Ripley Lucas, Taylor Thames, Jazmin F. Chestnut, Andrea L. DeMaria, Ashley Stoneburner

**Affiliations:** 1 Perinatal Data Center, March of Dimes, Arlington, Virginia; 2 Gillings School of Public Health, University of North Carolina, Chapel Hill; 3 March of Dimes, Arlington, Virginia

## Abstract

**Importance:**

Infant mortality, ie, death within the first year of life, serves as a critical health indicator.

**Objective:**

To evaluate the association of maternal residence in counties with no or limited access to maternity care with infant mortality overall and by maternal race and ethnicity and timing of death.

**Design, Setting, and Participants:**

This cross-sectional study used cohort-linked birth/infant death records for live births to US residents from 2017 to 2021, collected from the National Center for Health Statistics. Statistical analyses were conducted from July 2024 to June 2025.

**Exposures:**

Maternity care access by maternal county residence. Care access was categorized as none, low, moderate, or full based on availability of obstetric hospitals and birth centers, ratio of obstetric clinicians to births, and proportion of uninsured women aged 19 to 54 years.

**Main Outcome and Measures:**

The association between infant mortality and access to maternity care was assessed using multivariable log-binomial regression. Stratified analyses were conducted by timing of death (neonatal and postneonatal) and maternal race and ethnicity to assess for differences by subgroups.

**Results:**

A total of 18 682 916 live births were included (maternal age: 5 458 056 [29.2%] aged 30-34 years; maternal race and ethnicity: 4 426 077 [23.7%] Hispanic, 2 711 614 [14.5%] non-Hispanic Black, and 9 600 056 [51.4%] non-Hispanic White). Infant mortality rates (deaths per 1000 live births) increased as access decreased, with the highest rates in no-access counties (6.5 deaths per 10 000 live births) and the lowest in full-access counties (5.2 deaths per 1000 live births). The adjusted model found that infants in no-access counties had higher mortality risk compared with those in full-access counties (adjusted risk ratio, 1.14; 95% CI, 1.10-1.17; *P* < .001). When stratified by maternal race and ethnicity, non-Hispanic White infants in no-access counties had higher risk compared with non-Hispanic White infants in full-access counties (adjusted risk ratio, 1.20; 95% CI, 1.16-1.25). No significant differences were identified for other racial and ethnic groups. When stratified by timing of death, risk in the neonatal and postneonatal period was higher for infants in no-access counties compared with those in full-access counties (neonatal: adjusted risk ratio, 1.15; 95% CI, 1.11-1.19; postneonatal: adjusted risk ratio, 1.12; 95% CI, 1.06-1.17).

**Conclusions and Relevance:**

In this population-based cross-sectional study, infants in no-access counties had higher mortality risk than those with full access, persisting regardless of time of death. When examined by race and ethnicity, differences in mortality risk between living in a full access and no access county were observed among White infants only, indicating that unmeasured barriers may limit the protective effect of access for some racial and ethnic groups.

## Key Points

**Question:**

Is limited access to maternity care at the county level associated with increased risk of infant mortality?

**Findings:**

In this cross-sectional analysis of more than 18 million live births, the adjusted risk of infant mortality among those residing in counties with no access to maternity care was 14% higher than infants residing in counties with at least 2 hospitals or birth centers offering obstetric care and at least 60 obstetric clinicians per 10 000 births.

**Meaning:**

In this study, infant mortality risk was inversely associated with county-level access to maternity care, with the highest risk in counties with no access.

## Teaser

This cross-sectional study evaluates whether county-level access to maternity care in the United States is associated with rates of infant mortality overall, by race and ethnicity, and by timing of the death.

## Introduction

Infant mortality is a critical indicator of a nation’s health, reflecting the social, economic, and cultural conditions that shape the lives of infants and their caregivers. The infant mortality rate (IMR; number of infant deaths per 1000 live births) measures health care quality and broader societal well-being.^1^ In 2022, the US recorded its first statistically significant year-over-year increase in the IMR in 2 Decades, reversing a long-term trend of improvement.^2^ Between 2002 and 2022, the IMR declined nearly 20%, from 7.0 to 5.6 deaths per 1000 live births.^2,3^ However, these overall gains mask persistent and, in some cases, widening disparities across racial, ethnic, and geographic lines.

Not all groups have experienced equivalent reductions in infant mortality. The IMR among infants born to non-Hispanic American Indian or Alaska Native birthing people rose 5%, from 8.6 per 1000 live births in 2002 to 9.1 per 1000 live births in 2022. As a result, this group is the only one with a higher IMR in 2022 than in 2002. In contrast, the IMRs for infants born to non-Hispanic Black and White birthing people have decreased by more than 20% since 2002. However, the disparity has persisted, with infants born to non-Hispanic Black birthing people dying more than twice as often as those born to non-Hispanic White birthing people.^2,3^

The overall decrease in the US IMR masks historical and current disparities among states, as IMRs over the last 20 years have consistently remained highest in the South and Midwest regions.^2,3^ Furthermore, rural areas have significantly higher IMRs than urban areas, illustrating that geographical disparities persist within states with relatively low IMRs.^4,5,6^ Place is increasingly recognized as a driver of health outcomes, including infant mortality, partly due to the resources an individual can access within their community.^7^ Barriers to health care range from challenges resulting from what is known as structural urbanism, such as long travel distances and limited clinician availability, to systemic issues, including insurance enrollment difficulties and lack of culturally congruent care.^8,9,10,11,12^ These barriers are often compounded for rural communities of minoritized racial and ethnic groups, where the intersection of place and structural racism can further limit access and exacerbate disparities.^13^

Access to equitable, high-quality maternity care is essential across the continuum of pregnancy. Before pregnancy, obstetric clinicians can provide counseling about topics associated with infant mortality risk.^14,15,16^ During pregnancy, adequate prenatal care decreases the risk of infant mortality by providing protective interventions, such as the treatment of chronic conditions, education on preterm birth warning signs, maternal vaccination, and early detection of fetal complications.^14,17^ In the postpartum period, services like postpartum mental health screening and caregiver newborn education may play a role in preventing infant deaths.^14,18,19^ Research suggests that barriers to quality maternity care have a cascading effect, where those with limited access to care may be less prepared for pregnancy, receive inadequate prenatal care, and may potentially be at a higher risk of complications, including infant death.^20,21,22^

Despite the importance of equitable, high-quality care throughout pregnancy, much of the United States continues to face unmet maternal health needs. In counties lacking maternity care access, limited resources may contribute to persistent cycles of poor health outcomes.^23,24,25,26^ Previous research has identified relationships between individual-level factors and their association with infant mortality,^27,28,29,30^ but no known studies, to date, have explored the association between maternity care access designations and infant mortality.

This study investigates the association between infant mortality and access to maternity care, using March of Dimes’ maternity care access designations.^31^ We aim to determine whether infants born to birthing people in areas with no or limited access to maternity care are at an increased risk of death and whether risk varies by race and ethnicity or timing of death.

## Methods

### Study Population

This cross-sectional study was conducted using 2017 through 2021 National Center for Health Statistics (NCHS) cohort-linked birth/infant death files for all US states and the District of Columbia.^32^ This study followed the Strengthening the Reporting of Observational Studies in Epidemiology (STROBE) reporting guidelines. Solutions IRB approved the study and waived the requirement for informed consent for secondary data use.

### Primary Exposure

The primary exposure was county-level maternity care access designations developed by March of Dimes (Table 1). These designations categorized counties as maternity care deserts or low, moderate, or full access based on 3 factors: availability of obstetric hospitals and birth centers, ratio of obstetric clinicians to births, and proportion of uninsured women aged 19 to 54 years. For this analysis, we use the term *no-access counties* in place of maternity care deserts to improve clarity and acknowledge the limitations of the term deserts in this context.^33^ Counties classified as no-access had no hospitals or birth centers providing obstetric care and no obstetric clinicians. Low-access counties had fewer than 2 hospitals or birth centers offering obstetric care, fewer than 60 obstetric clinicians per 10 000 births, and 10% or more of women aged 19 to 54 years without insurance. Moderate-access counties met the same thresholds for clinicians and birthing facilities as low-access counties, but fewer than 10% of women aged 19 to 54 years were uninsured. Full-access counties had either 2 or more hospitals or birth centers offering obstetric care or at least 60 obstetric clinicians per 10 000 births.^34^

**Table 1.** County-Level Maternity Care Access Designations Developed by March of Dimes

Designation component^a^	Maternity care access designation			
	No access	Low access	Moderate access	Full access
Hospitals and birth centers offering obstetric care	0	<2	<2	≥2
Obstetric clinicians (OB-GYN, family physician, CNM/CM) per 10 000 births^b^	0	<60	<60	≥60
Proportion of women aged 19-54 y without health insurance	Any	≥10%	<10%	Any

Abbreviations: CNM/CM, certified nurse midwife/certified midwife; OB-GYN, obstetrician-gynecologist.

^a^ Family physicians were added to the access designation criteria in 2022. Birth center locations were incorporated beginning in 2020.

^b^ Includes only family physicians who report delivering babies.

### Outcome

The primary outcome was infant mortality, defined as death occurring within the first year of life. Death certificates for infants are linked to state birth records and weighted as part of the National Vital Statistics System.^35^ Causes of death were classified using the *International Statistical Classification of Diseases and Related Health Problems, Tenth Revision *(*ICD-10*), and determinations followed protocols outlined in the NCHS Handbook on Death Registration and Fetal Death Reporting.^36,37^ Subanalyses by cause of death included those resulting from the 5 leading causes of infant death (ie, unintentional injuries, complications of pregnancy, congenital anomalies, preterm birth or low birthweight, sudden infant death syndrome [SIDS]), and all other causes. Infant deaths were classified as neonatal (0-27 days after birth) or postneonatal (28 days to 1 year after birth).

### Covariates

Covariates were selected based on prior literature on risk factors for infant mortality, with the aid of a directed acyclic graph to inform confounder control and minimize bias.^38^ Maternal self-reported covariates included: age (categorized as <20, 20-24, 25-29, 30-34, 35-39, and ≥40 years), smoking in the 3 months before pregnancy (categorized as daily use of 1 or more cigarettes), education level (categorized as high school graduate or less and completing some college or more), and race and ethnicity. Hispanic ethnicity was classified first, while individuals who reported non-Hispanic ethnicity were further classified as American Indian or Alaska Native; Asian, Native Hawaiian, or Other Pacific Islander; Black; White; or more than 1 race. Other covariates included maternal payment source at the time of birth (Medicaid, private insurance, self-pay, or other), plurality (singleton or multiple births), infant birth year, and maternal health conditions (hypertension or diabetes, either prepregnancy or gestational). For this study, we refer to infants born to birthing people of race and ethnicity groups as infants of that group (eg, infants born to Black birthing people are described as Black infants).

### Statistical Analysis

Descriptive statistics were used to examine IMRs across covariates and leading causes of death. We conducted χ^2^ tests and standardized differences were calculated to assess differences in covariates across access categories. Standardized differences greater than 0.1 or less than −0.1 were used to indicate a meaningful difference between groups. We explored leading cause of death by maternal race and ethnicity. Log-binomial regression models were used to estimate crude and adjusted risk ratios (aRR) for infant mortality among infants born to birthing people residing in no-, low-, and moderate-access counties compared with full-access counties. The adjusted model included maternal age, maternal smoking, payment source, maternal race and ethnicity, plurality, education level, infant birth year, and maternal health conditions. *P* values less than .05 indicated statistical significance, and all tests were 2-tailed. Subgroup analyses by timing of death and maternal race and ethnicity were conducted to examine potential effect measure modification in the association between access and infant mortality. All cases had complete primary exposure and outcome data. Cases with missing covariate data (528 828 [2.8%]) were excluded from IMR calculations and regression analyses. All analytical variables were missing less than 1% of cases (maternal race and ethnicity, 172 568 [0.9%]; payment source, 125 625 [0.7%]; maternal smoking, 86 427 [0.5%]; and maternal health conditions, 19 166 [0.1%]), except for maternal education (261 195 [1.4%]). Infant birth year, maternal age, and plurality had no missing cases. Missingness patterns were evaluated for all analytical variables to assess likelihood and pairwise concordance.^39^ No significant pairwise concordance was observed between variables, with maternal race and ethnicity and education level missing in tandem most frequently (0.54%). Attributable risk was calculated to estimate the proportion of infant deaths potentially attributable to reduced maternity care access in no- and low-access counties.^40^ All statistical analyses were conducted using SAS version 9.4 (SAS Institute) from July 2024 to June 2025.

## Results

From 2017 through 2021, there were 18 682 916 live births (maternal age: 5 458 056 [29.2%] aged 30-34 years; maternal race and ethnicity: 4 426 077 [23.7%] Hispanic, 2 711 614 [14.5%] non-Hispanic Black, and 9 600 056 [51.4%] non-Hispanic White), with 718 143 (3.8%) to residents of no-access counties. An additional 1.45 million babies were born to residents of counties with low or moderate access to maternity care. Compared with residents of full-access counties, those in no-access areas were more often White, insured by Medicaid, and younger than 30 years and had lower educational attainment and higher rates of cigarette smoking (Table 2). Counties with less maternity care access had higher mortality rates due to congenital anomalies, SIDS, and unintentional injuries. Mortality rates from SIDS and unintentional injuries were 62.3% and 64.5% higher, respectively, in no-access counties than in full-access counties (Table 3).

**Table 2.** Study Population Characteristics by Maternity Care Designations

Characteristic	No access^a^		Low access^a^		Moderate access^a^		Full access, No. (%)^a^	Total, No. (%)	*P* value
	No. (%)	SD^b^	No. (%)	SD^b^	No. (%)	SD^b^			
Births									
Total, No.	718 143	NA	924 867	NA	521 293	NA	16 518 613	18 682 916	<.001
Birth year									
2017	148 579 (20.7)	0.003	188 351 (20.4)	−0.005	124 106 (23.8)	0.078	3 394 464 (20.6)	3 855 500 (20.6)	<.001
2018	147 470 (20.5)	0.008	186 909 (20.2)	0.000	122 689 (23.5)	0.081	3 337 287 (20.2)	3 794 355 (20.3)	
2019	148 130 (20.6)	0.016	198 916 (21.5)	0.038	104 478 (20.0)	0.002	3 298 526 (20.0)	3 750 050 (20.1)	
2020	143 207 (19.9)	0.018	193 658 (20.9)	0.042	100 639 (19.3)	0.002	3 178 552 (19.2)	3 616 056 (19.4)	
2021	130 757 (18.2)	−0.047	157 033 (17.0)	−0.079	69 381 (13.3)	−0.181	3 309 784 (20.0)	3 666 955 (19.6)	
Maternal race and ethnicity									
Hispanic	76 571 (10.7)	−0.380	184 215 (19.9)	−0.121	44 468 (8.5)	−0.5	4 120 823 (25.0)	4 426 077 (23.7)	<.001
Non-Hispanic AIAN	17 332 (2.4)	0.146	16 002 (1.7)	0.102	2884 (0.6)	−0.01	104 377 (0.6)	140 595 (0.8)	
Non-Hispanic ANHPI	5327 (0.7)	−0.334	15 975 (1.7)	−0.266	7796 (1.5)	−0.451	1 180 774 (7.2)	1 209 872 (6.5)	
Non-Hispanic Black	76 892 (10.7)	−0.128	130 027 (14.1)	−0.026	31 323 (6.0)	−0.296	2 473 372 (15.0)	2 711 614 (14.5)	
Non-Hispanic White	527 719 (73.5)	0.519	555 365 (60.1)	0.223	421 435 (80.8)	0.708	8 095 537 (49.0)	9 600 056 (51.4)	
Non-Hispanic >1 race	12 348 (1.7)	−0.041	20 324 (2.2)	−0.007	10 192 (2.0)	−0.024	379 270 (2.3)	422 134 (2.3)	
Missing	1954 (0.3)	−0.091	2959 (0.3)	−0.084	3195 (0.6)	−0.043	164 460 (1)	172 568 (0.9)	
Insurance									
Medicaid	358 748 (50.0)	0.179	455 159 (49.2)	0.164	211 981 (40.7)	−0.009	6 788 899 (41.1)	7 814 787 (41.8)	<.001
Private	301 365 (42.0)	−0.177	367 011 (39.7)	−0.224	266 901 (51.2)	0.009	8 383 078 (50.8)	9 318 355 (49.9)	
Self-pay	32 498 (4.5)	0.030	51 700 (5.6)	0.078	20 337 (3.9)	−0.001	647 969 (3.9)	752 504 (4.0)	
Other	20 552 (2.9)	−0.040	44 697 (4.8)	0.063	17 917 (3.4)	−0.007	588 479 (3.6)	671 645 (3.6)	
Missing	4980 (0.7)	0.003	6300 (0.7)	0.002	4157 (0.8)	0.015	110 188 (0.7)	125 625 (0.7)	
Plurality									
Singleton	694 831 (96.8)	0.003	895 379 (96.8)	0.007	503 924 (96.7)	−0.002	15 972 712 (96.7)	18 066 846 (96.7)	<.001
Multiples	23 312 (3.3)	−0.003	29488 (3.2)	−0.007	17369 (3.3)	0.002	545901 (3.3)	616070 (3.3)	
Maternal age, y									
<20	52 132 (7.3)	0.125	64 193 (6.9)	0.113	28 163 (5.4)	0.050	716 452 (4.3)	860 940 (4.6)	<.001
20-24	198 994 (27.7)	0.237	245 662 (26.6)	0.211	121 646 (23.3)	0.136	2 945 854 (17.8)	3 512 156 (18.8)	
25-29	236 094 (32.9)	0.103	294 342 (31.8)	0.080	173 016 (33.2)	0.110	4 648 941 (28.1)	5 352 393 (28.7)	
30-34	155 105 (21.6)	−0.194	209 368 (22.6)	−0.169	132 924 (25.5)	−0.101	4 961 659 (30.0)	5 459 056 (29.2)	
35-39	62 924 (8.8)	−0.221	91 529 (9.9)	−0.182	54 877 (10.5)	−0.162	2 642 269 (16.0)	2 851 599 (15.3)	
≥40	12 894 (1.8)	−0.114	19 773 (2.1)	−0.090	10 667 (2.1)	−0.097	603 438 (3.7)	646 772 (3.5)	
Maternal education									
≤High school graduate	349 581 (48.9)	0.246	445 906 (48.5)	0.237	216 834 (41.9)	0.102	6 046 190 (37.2)	7 058 511 (38.3)	<.001
≥Some college	365 689 (51.1)	−0.222	474 403 (51.6)	−0.215	300 383 (58.1)	−0.087	10 222 735 (62.8)	11 363 210 (61.7)	
Missing	2873 (0.4)	−0.114	4558 (0.5)	−0.102	4076 (0.8)	−0.069	249 688 (1.5)	26 1195 (1.4)	
Maternal smoking									
Yes, ≥1	119 527 (16.6)	0.315	111 898 (12.1)	0.188	90 062 (17.3)	0.332	1 099 085 (6.7)	1 420 572 (7.6)	<.001
No	594 811 (82.8)	−0.312	809 282 (87.5)	−0.182	428 931 (82.3)	−0.326	15 342 893 (92.9)	17 175 917 (91.9)	
Missing	3805 (0.5)	0.009	3687 (0.4)	−0.010	2300 (0.4)	−0.003	76 635 (0.5)	86 427 (0.5)	
Maternal health condition									
Yes	124 897 (17.4)	0.026	155 851 (16.9)	0.011	93 137 (17.9)	0.038	2 714 550 (16.5)	3 088 435 (16.5)	<.001
No	592 171 (82.6)	−0.027	768 187 (83.1)	−0.011	427 159 (82.1)	−0.040	13787798 (83.6)	15 575 315 (83.5)	
Missing	1075 (0.1)	0.015	829 (0.1)	−0.003	997 (0.2)	0.024	16 265 (0.1)	19 166 (0.1)	

Abbreviations: NA, not applicable; SD, standardized difference.

^a^ No-access county: a county with no obstetric hospitals, birth centers, or obstetric clinicians. Low-access county: a county with fewer than 60 obstetric clinicians per 10 000 live births, fewer than 2 obstetric hospitals or birth centers, and 10% or more of women aged 19 to 54 years uninsured. Moderate-access county: a county with fewer than 60 obstetric clinicians per 1000 birth, fewer than 2 obstetric hospitals or birth centers, and less than 10% of women aged 19 to 54 years uninsured. Full-access county: a county with more than 2 obstetric hospitals or birth centers or more than 60 obstetric clinicians per 10 000 live births.

^b^ SDs use full-access counties as the reference group.

**Table 3.** Infant Mortality Rates for Analytical Covariates by Maternity Care Designations, With Missing Data Removed

Characteristic	No access			Low access			Moderate access			Full access			Total		
	Deaths, No.	Births, No.	IMR^a^	Deaths, No.	Births, No.	IMR^a^	Deaths, No.	Births, No.	IMR^a^	Deaths, No.	Births, No.	IMR^a^	Deaths, No.	Births, No.	IMR^a^
Total	4583	705 033	6.5	5787	908 665	6.4	2864	510 216	5.6	83 356	16 030 174	5.2	96 590	18 154 088	5.3
Birth year															
2017	1016	145 783	7.0	1220	185 293	6.6	657	121 530	5.4	17 736	3 299 677	5.4	20 629	3 752 283	5.5
2018	906	145 061	6.2	1138	184 084	6.2	649	120 224	5.4	17 242	3 243 686	5.3	19 935	3 693 055	5.4
2019	959	145 245	6.6	1254	195 022	6.4	571	102 494	5.6	16 460	3 196 940	5.1	19 244	3 639 701	5.3
2020	883	140 612	6.3	1197	189 874	6.3	593	98 373	6.0	15 433	3 084 548	5.0	18 106	3 513 407	5.2
2021	819	128 332	6.4	978	154 392	6.3	394	67 595	5.8	16 485	3 205 323	5.1	18 676	3 555 642	5.3
Timing of death															
Neonatal	2774	705 033	3.9	3566	908 665	3.9	1843	510 216	3.6	54 257	16 030 174	3.4	62 440	18 154 088	3.4
Postneonatal	1809	705 033	2.6	2221	908 665	2.4	1021	510 216	2.0	29 099	16 030 174	1.8	34 150	18 154 088	1.9
Maternal race and ethnicity															
Hispanic	405	75 196	5.4	900	181 077	5.0	208	43 435	4.8	18 892	4 022 823	4.7	20 405	4 322 531	4.7
Non-Hispanic AIAN	158	16 549	9.5	139	15 596	8.9	19	2780	6.8	747	101 503	7.4	1063	136 428	7.8
Non-Hispanic ANHPI	24	5198	4.6	81	15 678	5.2	35	7659	4.6	4025	1 153 648	3.5	4165	1 182 183	3.5
Non-Hispanic Black	854	76 010	11.2	1359	128 657	10.6	316	30 766	10.3	24751	2 420 750	10.2	27 280	2 656 183	10.3
Non-Hispanic White	3045	519 934	5.9	3143	547 608	5.7	2211	415 530	5.3	32588	7 960 144	4.1	40 987	9 443 216	4.3
Non-Hispanic >1 race	97	12 146	8.0	165	20 049	8.2	75	10 046	7.5	2353	371 306	6.3	2690	413 547	6.5
Insurance															
Medicaid	2818	353 980	8.0	3415	449 564	7.6	1482	208 218	7.1	45 394	6 614 861	6.9	53 109	7 626 623	7
Private	1384	299 066	4.6	1766	364 080	4.9	1151	264 409	4.4	31 174	8 211 291	3.8	35 475	9 138 846	3.9
Self-pay	226	31 757	7.1	356	50 766	7.0	135	19 881	6.8	3711	629 356	5.9	4428	731 760	6.1
Other	155	20 230	7.7	250	44 255	5.6	96	17 708	5.4	3077	574 666	5.4	3578	656 859	5.4
Plurality															
Singleton	4055	682 152	5.9	5003	879 685	5.7	2471	493 162	5.0	72 181	15 502 463	4.7	83 710	17 557 462	4.8
Multiples	528	22 881	23.1	784	28 980	27.1	393	17 054	23.0	11 175	527 711	21.2	12 880	596 626	21.6
Maternal age, y															
<20	436	51 129	8.5	543	62 993	8.6	254	27 516	9.2	5863	695 710	8.4	7096	837 348	8.5
20-24	1306	195 476	6.7	1666	241 599	6.9	775	119 162	6.5	18 607	2 864 124	6.5	22 354	3 420 361	6.5
25-29	1399	232 119	6.0	1654	289 470	5.7	877	169 604	5.2	22 855	4 519 943	5.1	26 785	5 211 136	5.1
30-34	878	152 260	5.8	1127	205 680	5.5	594	130 159	4.6	20 542	4 819 152	4.3	23 141	5 307 251	4.4
35-39	442	61 485	7.2	607	89 662	6.8	287	53 465	5.4	11 802	2 554 535	4.6	13 138	2 759 147	4.8
≥40	122	12 564	9.7	190	19 261	9.9	77	10 310	7.5	3687	576 710	6.4	4076	618 845	6.6
Maternal education															
≤High school graduate	2732	343 555	8.0	3362	439 438	7.7	1588	213 151	7.5	42 071	5 942 820	7.1	49 753	6 938 964	7.2
≥Some college	1851	361 478	5.1	2425	469 227	5.2	1276	297 065	4.3	41 285	1 008 7354	4.1	46 837	11 215 124	4.2
Maternal smoking															
Yes, ≥1	1116	117 768	9.5	1104	110 259	10.0	759	88 636	8.6	10567	1 077 571	9.8	13 546	1 394 234	9.7
No	3467	587 265	5.9	4683	798 406	5.9	2105	421 580	5.0	72 789	14 952 603	4.9	83 044	16 759 854	5
Maternal health condition															
Yes	831	123 026	6.8	1018	153 458	6.6	479	91 620	5.2	14 488	2 642 155	5.5	16 816	3 010 259	5.6
No	3752	582 007	6.4	4769	755 207	6.3	2385	418 596	5.7	68 868	13 388 019	5.1	79 774	15 143 829	5.3
Leading causes of death															
Unintentional injuries	360	705 033	0.5	436	908 665	0.5	230	510 216	0.5	4977	16 030 174	0.3	6003	18 154 088	0.3
Complications of pregnancy	167	705 033	0.2	266	908 665	0.3	138	510 216	0.3	4955	16 030 174	0.3	5526	18 154 088	0.3
Congenital anomalies	1017	705 033	1.4	1218	908 665	1.3	688	510 216	1.3	17 110	16 030 174	1.1	20 033	18 154 088	1.1
Prematurity or low birthweight	620	705 033	0.9	785	908 665	0.9	435	510 216	0.9	13 267	16 030 174	0.8	15 107	18 154 088	0.8
SIDS	396	705 033	0.6	443	908 665	0.5	198	510 216	0.4	5528	16 030 174	0.3	6565	18 154 088	0.4
All other causes	2023	705 033	2.9	2639	908 665	2.9	1175	510 216	2.3	37 519	16 030 174	2.3	43 356	18 154 088	2.4

Abbreviations: AIAN, American Indian or Alaska Native; ANHPI, Asian, Native Hawaiian, or Other Pacific Islander; IMR, infant mortality rate; SIDS, sudden infant death syndrome.

^a^ IMR was measured per 1000 live births.

Among the analytical sample, the national IMR for births from 2017 to 2021 was 5.3 deaths per 1000 live births. An inverse association was observed between access and IMRs: the highest IMR was in no-access counties at 6.5 deaths per 1000 live births and decreased by access designation, reaching the lowest IMR at 5.2 deaths per 1000 live births in full-access counties (Table 3). Infants residing in no-access counties were significantly more likely to die compared with those in full-access counties. In unadjusted models, the risk of infant death in no-access counties was significantly higher (RR, 1.25, 95% CI, 1.21-1.29; *P* < .001) than in full-access counties. After adjustment, the RR decreased to 1.14 (95% CI, 1.11-1.17; *P* < .001) (Table 4). In low-access counties, the adjusted aRR for infant death was 1.12 (95% CI, 1.09-1.15; *P* < .001) (Table 4). Nearly 1 in 8 deaths in no-access counties and 1 in 9 deaths in low-access counties were potentially attributable to lack of access to maternity care.

**Table 4.** Risk of Infant Mortality Across Maternity Care Access Designations Compared With Full-Access Counties

Stratification level	No-access vs full-access			Low-access vs full-access			Moderate-access vs full-access		
	Crude RR (95% CI)	Adjusted RR (95% CI)^a^	*P* value^b^	Crude RR (95% CI)	Adjusted RR (95% CI)^a^	*P* value^b^	Crude RR (95% CI)	Adjusted RR (95% CI)^a^	*P* value^b^	
Overall	1.25 (1.21-1.29)	1.14 (1.10-1.17)	<.001	1.22 (1.19-1.26)	1.12 (1.09-1.15)	<.001	1.08 (1.04-1.12)	1.08 (1.04-1.12)	<.001	
Timing of death										
Neonatal	1.16 (1.12-1.21)	1.15 (1.11-1.19)	<.001	1.16 (1.12-1.20)	1.11 (1.08-1.15)	<.001	1.07 (1.02-1.12)	1.14 (1.09-1.19)	<.001	
Postneonatal	1.41 (1.35-1.48)	1.12 (1.06-1.17)	<.001	1.35 (1.29-1.41)	1.12 (1.07-1.17)	<.001	1.10 (1.04-1.17)	0.99 (0.93-1.06)	.86	
Maternal race/ethnicity										
Hispanic	1.15 (1.04-1.27)	1.07 (0.97-1.18)	.17	1.06 (0.99-1.13)	1.00 (0.94-1.07)	.96	1.02 (0.89-1.17)	0.98 (0.85-1.12)	.70	
Non-Hispanic AIAN	1.30 (1.09-1.54)	1.16 (0.98-1.38)	.08	1.21 (1.01-1.45)	1.18 (0.98-1.41)	.08	0.93 (0.59-1.46)	0.84 (0.53-1.32)	.45	
Non-Hispanic ANHPI	1.32 (0.89-1.97)	1.17 (0.78-1.74)	.45	1.48 (1.19-1.84)	1.37 (1.10-1.71)	.005	1.31 (0.94-1.83)	1.24 (0.89-1.73)	.20	
Non-Hispanic Black	1.10 (1.03-1.18)	1.00 (0.94-1.07)	.94	1.03 (0.98-1.09)	0.99 (0.93-1.04)	.64	1.00 (0.90-1.12)	0.94 (0.84-1.05)	.26	
Non-Hispanic White	1.43 (1.38-1.48)	1.20 (1.16-1.25)	<.001	1.40 (1.35-1.45)	1.22 (1.18-1.27)	<.001	1.3 (1.25-1.36)	1.14 (1.09-1.19)	<.001	
Non-Hispanic >1 race	1.26 (1.03-1.54)	1.08 (0.88-1.32)	.48	1.30 (1.11-1.52)	1.16 (0.99-1.36)	.07	1.18 (0.94-1.48)	0.98 (0.78-1.23)	.85	

Abbreviations: AIAN, American Indian or Alaska Native; ANHPI, Asian, Native Hawaiian, or Other Pacific Islander; RR, risk ratio.

^a^ Adjusted model includes maternal age, maternal smoking, payment source, maternal race and ethnicity, plurality, maternal education, maternal health condition, and birth year. Reference categories for adjusted models are maternal age, 30 to 34 years; maternal smoking, nonsmoker; payment source, private insurance; maternal race and ethnicity, non-Hispanic White; plurality, singleton; maternal education, some college or more; maternal health conditions, no; birth year, 2017.

^b^ *P* value for adjusted model.

### Differences by Maternal Race and Ethnicity

No-access counties had a higher proportion of White infants (527 719 of 718 143 [73.5%]) than full-access counties (8 095 537 of 16 518 613 [49.0%]) (*P* < .001). However, American Indian or Alaska Native infants were disproportionately represented in no-access counties (17443 [2.4%] compared with 104 377 [0.6%] in full-access counties) (Table 2). American Indian or Alaska Native and Black infants in no-access counties experienced the highest IMRs at 9.5 and 11.2 deaths per 1000 live births, respectively. Racial disparities persisted in full-access counties, where the IMR for Black infants was more than double the IMR for White infants (10.2 and 4.1 deaths per 1000 live births, respectively) (Table 4).

The distribution of the leading causes of death varied by race and ethnicity. Deaths attributed to preterm birth or low birthweight accounted for the largest proportion among Black infants (20.6% of deaths), whereas congenital abnormalities represented the largest proportion of deaths in all other groups. The proportion of deaths from SIDS and unintentional injuries was highest among American Indian or Alaska Native infants, with each accounting for approximately 10% of deaths (eTable in Supplement 1).

White infants in no-access counties experienced a significantly higher risk of death, with an aRR of 1.20 (95% CI, 1.16-1.25; *P* < .001). Similarly, Asian, Native Hawaiian, or Other Pacific Islander infants in low-access counties had a significantly elevated aRR of 1.37 (95% CI, 1.10-1.71; *P* = .005). For American Indian or Alaska Native infants, aRRs for infant mortality in no- and low-access counties were elevated but not statistically significant. Adjusted RRs for Black and Hispanic infants indicated no significant variation in risk for these groups (Table 4).

### Differences by Timing of Death

Neonatal deaths accounted for nearly two-thirds (62 440 of 96 590 [65.8%]) of all deaths, with a smaller proportion in no-access counties (2774 of 4583 [61.3%]). Both neonatal and postneonatal deaths were inversely associated with access, with postneonatal deaths exhibiting larger relative disparities between designations. The postneonatal IMR was 2.6 deaths per 1000 live births in no-access counties compared with 1.8 per 1000 live births in full-access counties, while the neonatal IMR was 3.9 per 1000 live births in no-access counties and 3.4 per 1000 live births in full-access counties (Table 3).

Risks were elevated for neonatal and postneonatal deaths in no- and low-access counties. For infants in no-access counties, the unadjusted RRs were higher at 1.16 (95% CI, 1.12-1.21; *P* < .001) for neonatal deaths and 1.41 (95% CI, 1.35-1.48; *P* < .001) for postneonatal deaths, compared with full-access counties. After adjustment, the risk did not meaningfully differ between neonatal and postneonatal infants, as the risk of neonatal death in no-access counties remained consistent at 1.15 (95% CI, 1.11-1.19; *P* < .001) and the risk of postneonatal death decreased to 1.12 (95% CI, 1.06-1.17; *P* < .001).

## Discussion

In this US population-based cross-sectional analysis, we found that county-level access to maternity care was associated with infant mortality risk. Significantly higher risks were observed in no-, low-, and moderate-access counties compared with full-access counties, with the highest risk in no-access counties. Our analysis explores the association between access to maternity care and infant mortality using maternity care access designations and supports similar findings using proxy measures of access, such as rurality or prenatal care utilization.^5,6,8,20,21,22^ We found the risk of infant mortality was significantly higher in counties with less access within the overall study population; however, we did not observe this for all racial and ethnic groups in stratified analyses. White infants faced significantly higher mortality risks in no-access counties, as well as in low- and moderate-access counties, compared with full-access counties. While not significant in our adjusted model, we observed a similar association among American Indian or Alaska Native infants. In addition, Asian, Native Hawaiian, or Other Pacific Islander infants in low-access counties experienced significantly elevated infant mortality risk compared with full-access counties. This contrasts with Black and Hispanic infants, whose risk remained unchanged regardless of access designation. American Indian or Alaska Native and Black infants experienced persistently high rates overall that were 1.8 and 2.4 times the rate of White infants, respectively.

The racial and ethnic compositions of no- and full-access counties differed substantially, as nearly three-quarters of infants in no-access counties were White, compared with slightly less than half in full-access counties. All other racial and ethnic groups, except American Indian or Alaska Native, represented a smaller share of births in no-access counties compared with full-access counties. Additionally, distribution of cause of death differed between racial and ethnic groups. For instance, preterm birth/low birthweight deaths accounted for a larger proportion of deaths among Asian, Native Hawaiian, or Other Pacific Islander, Black, and Hispanic infants compared with White infants. In contrast, the proportion of deaths among White infants attributed to preterm birth has decreased over the last decade, leading to higher proportions of deaths from congenital malformations, SIDS, and unintentional injuries.^41,42,43^ This is notable as the latter-mentioned causes can be modifiable through access to health care before and during pregnancy, early detection, and education, compared with preterm birth, which is more directly modifiable through clinical care received in the perinatal period.^44,45,46,47^

Quality of care received during pregnancy and at birth, as well as access to support services, such as lactation, home visiting, and safe sleep programs, likely influence infant mortality risk. These elements may partially explain the association between access and infant mortality, as risk was elevated in both neonatal and postnatal periods. Our findings suggest that counties with no- or low-access may be affected across the continuum of infancy: lower quality of care may reduce neonatal survival, while lack of support services may impact caregiver knowledge and practices associated with infant survival.^48,49,50,51^ However, because our analysis was national, there are likely counties where elevated risk stems primarily from one, rather than both, time of death periods. Perinatal periods of risk analyses could therefore more precisely describe the burden of excess mortality and identify opportunities for prevention within no- and low-access communities.

The previously discussed elements require further consideration when examined within the context of race and ethnicity, as the absence of significant differences across access levels for American Indian or Alaska Native, Black, and Hispanic infants likely reflects intersecting factors. Research suggests that even when care is available, these populations are more likely to encounter lower-quality services, implicit bias, cultural discordance, or disrespectful treatment, limiting the benefits of access alone.^52,53,54^ Hospitals serving higher proportions of historically marginalized patients have been found to have fewer resources and lower capacity to implement evidence-based interventions.^55,56,57,58,59^ Socioeconomic inequities, such as financial security, housing stability, and transit barriers, persist regardless of the geographical availability of hospitals and clinicians and may further reduce the ability to receive care in these populations.^60,61,62^ The association between no-access residence and higher risk among White infants, but not among groups with the highest mortality rates, suggests that while access is fundamental for all infants, it alone cannot address the specific needs of historically marginalized populations.

Our findings reveal that infants born in no- and low-access counties are at a heightened risk for infant mortality. These counties share similar sociodemographic compositions and county-level characteristics when compared with counties with greater access to maternity care, often being rural areas with predominantly White populations, high rates of Medicaid coverage, and lower educational attainment. Limited access to care, compounded by individual- and community-level risk factors, creates significant challenges and presents an opportunity to implement evidence-based strategies across the continuum of infancy. Moreover, racial and ethnic disparities in infant mortality persist in the United States despite overall improvements, and the findings of this analysis suggest that there is still much to improve, even in areas with full access to care. Improved Medicaid reimbursement for rural hospitals, enhanced perinatal regionalization and telehealth, and stronger care networks can help reduce the risk of neonatal morbidity and mortality.^63,64,65^ For postneonatal infants, evidence-based prevention programs, such as home visiting, skill-building educational workshops, and community-led parental education, can prevent SIDS and unintentional injuries.^66,67,68^ Community engagement and perinatal workforce diversification are essential for improving infant health in high-risk areas and to address inequities by race and ethnicity.^69,70^

### Strengths and Limitations

A major strength of this analysis is that it includes all deaths among infants born in the United States from 2017 to 2021, thus creating a nationally representative, population-level dataset. More than 98% of birth and death records within the population were linked, increasing confidence in correlative findings.

This study also has limitations. The primary limitation of this analysis is the evolution of the definition of maternity care access over time, as changes in definition criteria and data sources may introduce misclassification bias. Furthermore, historical access designations may underestimate obstetric clinician availability, particularly in rural communities where family physicians often deliver maternity care. Additionally, hospital survey nonresponse may omit some obstetric hospitals. These limitations may overestimate the association between lack of maternity care and increased infant mortality risk. Factors such as caregiver behaviors, environmental exposures, and characteristics of the delivery hospital, including clinical practices and quality of care, remain unmeasured. The latter are particularly notable, as both can confound at the health care system level, where differences in practice and quality may be associated with geography as much as economics and policies.^71^ Furthermore, systematic inconsistencies and missingness in vital records, particularly regarding maternal information, may introduce bias and underestimate associations in our stratified, adjusted model. Additionally, the results reflect national averages and do not account for differences between states. State-level analyses may uncover important variations not reflected in this analysis.

## Conclusions

This national cross-sectional study found that limited access to maternity care was associated with infant mortality, with infants in no- and low-access counties facing the greatest risks. However, this association varied by race and ethnicity, revealing deeper structural inequities that shape infant survival beyond health care availability. American Indian or Alaska Native and Black infants experienced the highest mortality rates, regardless of access level. These elevated rates underscore the impact of historical marginalization and systemic racism on health outcomes, transcending the availability of care. Additionally, increased mortality risk persisted across infancy, which presents multiple opportunities for prevention throughout the neonatal and postneonatal periods. Expanding access is necessary but insufficient; solutions must address the broader social and economic conditions that shape maternal and infant health.

## Notes

Supplement 1. **eTable. **Infant Population for Cause of Death by Maternal Race and Ethnicity

Supplement 2.
Data Sharing Statement
