# Supplementary material for: Maternity Care Access and Infant Mortality
Source: JAMA Netw Open. 2025 Nov 11;8(11):e2542831. doi: 10.1001/jamanetworkopen.2025.42831 (PMC12606374; doi:10.1001/jamanetworkopen.2025.42831)
Supplement: Supplement 1. — eTable. Infant Population for Cause of Death by Maternal Race and Ethnicity [file jamanetwopen-e2542831-s001.pdf]

Supplemental Online Content

Lucas R, Thames T, Chestnut JF, DeMaria AL, Stoneburner A. Maternity care access and infant mortality. *JAMA Netw Open*. 2025;8(10):e2542831. doi:10.1001/jamanetworkopen.2025.42831

eTable. Infant Population for Cause of Death by Maternal Race and Ethnicity

This supplemental material has been provided by the authors to give readers additional information about their work.

© 2025 Lucas R et al. *JAMA Network Open*.

eTable. Infant Population for Cause of Death by Maternal Race and Ethnicity

	Maternal Race and Ethnicity, Infants, No. (%)						
Cause of death	Hispanic	Non-Hispanic, American Indian/Alaska Native	Non-Hispanic, Asian/Native Hawaiian or Other Pacific Islander	Non-Hispanic, Black	Non-Hispanic, White	Non-Hispanic, >1 race	Total
Accidents	839 (4.1)	103 (9.7)	137 (3.3)	1780 (6.5)	2888 (7.0)	256 (9.5)	6003 (6.2)
Complications of pregnancy	1341 (6.6)	44 (4.1)	310 (7.4)	1769 (6.5)	1918 (4.7)	144 (5.4)	5526 (5.7)
Congenital anomalies	5074 (24.9)	185 (17.4)	869 (20.9)	3723 (13.6)	9750 (23.8)	432 (16.1)	20033 (20.7)
Prematurity/low birth weight	3197 (15.7)	110 (10.3)	749 (18.0)	5614 (20.6)	5069 (12.4)	368 (13.7)	15107 (15.6)
Sudden infant death syndrome	971 (4.8)	107 (10.1)	137 (3.3)	2121 (7.8)	2977 (7.3)	252 (9.4)	6565 (6.8)
All other causes	8983 (44.0)	514 (48.4)	1963 (47.1)	12273 (45.0)	18385 (44.9)	1238 (46.0)	43356 (44.9)

© 2025 Lucas R et al. *JAMA Network Open*.
