# Supplementary material for: Maternity Care Access and Infant Mortality
Source: JAMA Netw Open. 2025 Nov 11;8(11):e2542831. doi: 10.1001/jamanetworkopen.2025.42831 (PMC12606374; doi:10.1001/jamanetworkopen.2025.42831)
Supplement: Supplement 2. — Data Sharing Statement [file jamanetwopen-e2542831-s002.pdf]

Data Sharing Statement

Lucas. Maternity Care Access and Infant Mortality. *JAMA Netw Open*. Published November 11, 2025. doi:10.1001/jamanetworkopen.2025.42831

Data
Data available: Yes
Data types: Data (not involving human participants), Data dictionary
How to access data: Contact author at rlucas@marchofdimes.org
When available: With publication

Supporting Documents
Document types: None

Additional Information
Who can access the data: Researchers whose proposed use of the data has been approved
Types of analyses: Public health research
Mechanisms of data availability: With investigator support and a signed data access agreement
Any additional restrictions: Maternity care access designations available upon request. Requests for infant data must be made directly to NCHS.
